# Molecular mechanisms of phytohormone ABA-regulated anthocyanin biosynthesis in grape berry: an epigenetic dual-gating hypothesis within a five-layer regulatory framework

**DOI:** 10.3389/fmolb.2026.1761085

**Published:** 2026-03-12

**Authors:** Yao Wang, Xiaohan Duan, Rui Xu, Huimin Huang

**Affiliations:** College of Environment and Ecology, Henan Vocational College of Water Conservancy and Environment, Zhengzhou, China

**Keywords:** abscisic acid, anthocyanin, DNA methylation, epigenetic regulation, grape berry, histone modification

## Abstract

Anthocyanin biosynthesis in grape berries is a complex process regulated by the phytohormone abscisic acid, yet the molecular mechanisms underlying cultivar-specific responses and environmental memory remain poorly understood. This review proposes an “epigenetic dual-gating” working hypothesis that positions DNA methylation and histone modifications as two sequential decision checkpoints hypothesized to govern ABA signal conversion into transcriptional output. The first gate, DNA methylation, determines transcription factor accessibility, with methylation levels classifying cultivars into three states: locked in non-pigmented cultivars with greater than 70% methylation, partially open in pink cultivars with 25%–70% methylation, and fully open in red cultivars with less than 25% methylation. The second gate, histone modifications, is hypothesized to determine chromatin permissiveness through the balance between activating marks such as H3K9ac and H3K4me3 and repressive marks such as H3K27me3 and H3K9me2. Both gates must be simultaneously open for VvMYBA activation and anthocyanin synthesis, although direct characterization of histone states at the VvMYBA locus in grape remains to be performed. This mechanism is further integrated into a five-layer regulatory framework encompassing ABA homeostasis, signal transduction, epigenetic gating, transcriptional network, and metabolic execution. The epigenetic layer is repositioned as an active decision-making center rather than a passive recorder, providing unified explanations for cultivar differences, environmental memory, and signal specificity. While mechanistic connections between ABA signaling and epigenetic modifications require further experimental validation, this conceptual framework integrates current knowledge, generates testable predictions, and offers theoretical foundations for epigenetic interventions in grape quality regulation under climate change scenarios.

## 1 Introduction

Grape berries exhibit one of the most striking color spectra in nature, ranging from pale green or golden yellow through pink to deep red and near-black purple. This remarkable diversity reflects not merely aesthetic variation but fundamental differences in anthocyanin biosynthesis pathways—metabolites that confer both pigmentation and significant health-promoting properties. A seminal study in 2004 revealed that the transition from red to non-pigmented grapes (such as pale green or golden varieties including Chardonnay and Sultanina) results from the insertion of a retrotransposon named Gret1 into the promoter region of *VvMYBA1*, effectively silencing anthocyanin synthesis capacity in these cultivars (Kobayashi et al., 2004). However, numerous intermediate phenotypes exist between these color extremes that cannot be explained by simple gene loss alone. For instance, the pink cultivar Benitaka maintains 68% DNA methylation in the 3′LTR region of the retrotransposon within the *VvMYBA1* regulatory region, whereas the deeply colored cultivar Brazil exhibits only 22% methylation at the same locus (Azuma and Kobayashi, 2022). This suggests that epigenetic modifications create a continuous spectrum of color expression rather than a simple genetic switch.

Such natural variation has acquired urgent practical significance against the backdrop of accelerating climate change. The 2024 European heatwave provided a cautionary example: vineyards across the Mediterranean experienced berry temperatures exceeding 40 °C during the critical véraison period, resulting in substantial anthocyanin loss and compromised wine color and quality. Notably, different cultivars exhibited marked heterogeneity in their responses to identical heat stress. Pinot Noir, renowned for producing elegant red wines, demonstrates extreme temperature sensitivity, with sustained high-temperature exposure nearly abolishing anthocyanin accumulation; in contrast, Merlot maintains considerable pigment deposition under similar conditions (de Rosas et al., 2022). These differential responses extend beyond immediate metabolic capacity. Recent evidence indicates that heat exposure triggers persistent epigenetic memory through modifications such as histone H3 lysine 27 trimethylation (H3K27me3), potentially affecting subsequent growing seasons (Nishio et al., 2024). Consequently, understanding the molecular basis of cultivar-specific environmental responses has evolved from academic curiosity to an urgent agricultural imperative, particularly as viticulture confronts unprecedented climatic fluctuations.

The phytohormone abscisic acid (ABA) has emerged as the master regulator of this complex developmental transition in non-climacteric fruits such as grape. Unlike climacteric species (e.g., tomato) that rely on ethylene to coordinate ripening, grape berries depend primarily on ABA to initiate and orchestrate the dramatic physiological changes occurring at véraison, including anthocyanin biosynthesis, sugar accumulation, and berry softening (Wheeler et al., 2009; Pilati et al., 2017). Multiple lines of evidence support the central role of ABA: endogenous ABA concentrations rise sharply at ripening initiation, exogenous ABA treatment accelerates véraison and anthocyanin accumulation in a dose-dependent manner, and ABA biosynthesis mutants display severely impaired berry ripening (Zhang et al., 2009). This hormonal signal must be understood within the broader context of non-climacteric fruit development, wherein the ripening process is irreversible and independent of autocatalytic ethylene production (Perotti et al., 2023), rendering the precision and reliability of ABA-mediated developmental switching particularly critical.

The molecular framework linking ABA perception to anthocyanin accumulation has been progressively established through three major waves of discovery spanning nearly 3 decades. The foundational period (1998–2004) established the biosynthetic machinery, beginning with Ford’s cloning of grape UDP-glucose:flavonoid 3-O-glucosyltransferase (*VvUFGT*)—the enzyme catalyzing a key step in anthocyanin biosynthesis (Hall et al., 2012)—followed by systematic characterization of flavonoid pathway gene expression in colored *versus* non-pigmented cultivars. The watershed moment came in 2004 when Kobayashi identified *VvMYBA1* and *VvMYBA2* as the master transcriptional regulators directly activating anthocyanin structural genes, while simultaneously discovering that the Gret1 insertion explains the genetic basis of non-pigmented grapes (Kobayashi et al., 2004). A breakthrough in 2009 saw two independent research groups simultaneously identify the pyrabactin resistance/PYR1-like (PYR/PYL) family as intracellular ABA receptors that release SNF1-related protein kinase 2 (SnRK2) to phosphorylate downstream transcription factors by inhibiting type 2C protein phosphatases (PP2Cs) (Ma et al., 2009)—a paradigm-shifting mechanism rapidly confirmed in grape through characterization of VvPYL1 and related family members (Li et al., 2012; Boneh et al., 2012b). Research from 2015 to 2020 further elucidated the transcriptional network architecture: Nicolas demonstrated that ABA-responsive element binding factor 2 (*VvABF2*) serves as a key transcriptional activator during berry ripening (Nicolas et al., 2014), while Hichri and colleagues clarified how *VvMYBA* proteins cooperate with *VvMYC1* within the MYB-bHLH-WD40 (MBW) transcriptional complex to activate *VvUFGT* and other anthocyanin biosynthesis genes (Hichri et al., 2010). Together, these findings construct an elegant linear model: ABA → PYR/PP2C/SnRK2 → ABF → *VvMYBA* → anthocyanin structural genes.

Nevertheless, this sophisticated molecular framework confronts three persistent empirical paradoxes that expose fundamental gaps in mechanistic understanding. The most immediate puzzle lies in the critical “signaling black box” between SnRK2 kinase activation and *VvMYBA* transcriptional induction. Although ABF transcription factors represent obvious candidates for bridging this gap, chromatin immunoprecipitation studies have failed to demonstrate direct ABF binding to the VvMYBA promoter, suggesting either technical limitations precluding detection or the existence of undiscovered intermediate steps. The molecular basis of inter-cultivar variation constitutes another unresolved enigma. While 68% methylation in Benitaka produces a pink phenotype and 22% methylation in Brazil yields deep red (Azuma and Kobayashi, 2022), no direct evidence exists for how ABA signaling regulates these epigenetic differences, although recent studies hint that ABA treatment can alter methylation patterns of ripening-related genes (Li et al., 2024). The mechanistic basis of environmental memory represents yet another challenge for current understanding. High temperature not only suppresses current anthocyanin synthesis but also establishes long-term repressive states through histone modifications such as *VvHDAC19*-mediated deacetylation (Jia et al., 2023) and heat stress-induced H3K27me3 (Nishio et al., 2024); however, how these epigenetic changes integrate with ABA signaling remains elusive.

These three knowledge gaps point toward a common hidden regulatory layer: epigenetic modifications may not merely passively record developmental states but actively “gate” the conversion of ABA signals into transcriptional responses. This review proposes an “epigenetic dual-gating” hypothesis that positions DNA methylation and histone modifications as two sequential decision checkpoints—only when both gates are open can VvMYBA be effectively activated. This epigenetic gating mechanism provides a unified explanation for all three paradoxes: cultivar differences arise from genetic variation in gating states, environmental memory is achieved through gating plasticity, and the signaling “black box” essentially represents an unknown pathway through which ABA regulates epigenetic enzyme recruitment. This review further integrates this mechanism into a five-layer network framework (ABA homeostasis–signal transduction–epigenetic gating–transcriptional network–metabolic pathway), positioning the epigenetic layer as an active decision-making center rather than a passive intermediary. This comprehensive perspective not only addresses critical knowledge gaps but also provides the theoretical foundation for epigenetic interventions in grape quality regulation under climate change scenarios. We acknowledge that direct experimental evidence linking ABA signaling to epigenetic enzyme recruitment in grape remains incomplete. Nevertheless, articulating this working hypothesis serves important scientific functions: it integrates previously disconnected observations (the methylation-phenotype correlation, persistent heat stress effects, and absent ABF-VvMYBA binding), generates specific testable predictions (such as ABA-induced VvROS1 enrichment at the VvMYBA locus), and builds upon mechanistically validated pathways in Arabidopsis and tomato. Throughout this review, we explicitly distinguish between conclusions supported by direct grape evidence and those inferred from cross-species studies.

## 2 ABA homeostasis

**FIGURE 1:**
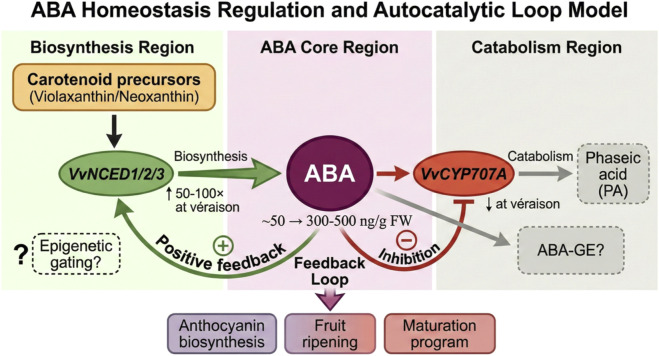
ABA homeostasis regulation and autocatalytic loop model.

The central role of abscisic acid in grape berry ripening depends on precise spatiotemporal regulation of its concentration. As illustrated in Figure 1, this dynamic equilibrium involves three functional modules: a biosynthetic compartment centered on *VvNCED*, an ABA core zone serving as a signal integration hub, and a catabolic compartment centered on *VvCYP707A*. These three modules form a self-regulatory network through positive and negative feedback loops, establishing an adjustable input system responsive to both developmental signals and environmental cues.

### 2.1 Biosynthesis via NCED

ABA biosynthesis proceeds through the carotenoid-derived pathway, wherein 9-cis-epoxycarotenoid dioxygenase (NCED) catalyzes the rate-limiting step, cleaving 9′-cis-violaxanthin or 9′-cis-neoxanthin to produce xanthoxin, which is subsequently converted to ABA through two oxidation steps. The grape genome encodes *VvNCED1*, *VvNCED2*, and *VvNCED3*, which play non-redundant roles during berry development (Young et al., 2012). Transcriptional profiling has revealed that *VvNCED1* exhibits basal expression prior to véraison, whereas *VvNCED2* and *VvNCED3* are sharply upregulated at véraison, with expression levels increasing 50- to 100-fold compared to young berries—dynamics that precisely correspond to the surge in endogenous ABA content from approximately 50 ng/g fresh weight to 300–500 ng/g (Speirs et al., 2013). This transcriptional activation is temporally synchronized with anthocyanin synthesis initiation and spatially localized predominantly in skin tissues.

Carotenoid precursors provide the metabolic foundation for *NCED* enzymatic activity. Grape berries accumulate substantial quantities of β-carotene, lutein, and violaxanthin during the green stage as a substrate pool for ABA synthesis; the decline in carotenoid content at véraison mirrors ABA accumulation (Young et al., 2012). However, significant gaps remain regarding upstream mechanisms governing *NCED* transcriptional regulation. Although sugar signaling, water stress, and hormonal crosstalk are known to influence *VvNCED* expression, transcription factors that directly bind to its promoter have yet to be identified. This knowledge gap limits mechanistic understanding of inter-cultivar differences in NCED expression—why do certain cultivars exhibit lower *VvNCED* induction amplitude, resulting in delayed or insufficient ABA accumulation? Comparative transcriptomic studies have demonstrated significant divergence in expression patterns and stress-response sensitivity among *VvNCED* family members across different organs, suggesting potential involvement of organ-specific promoter elements and epigenetic modifications (Rattanakon et al., 2016).

### 2.2 Catabolism and feedback inhibition

Precise ABA regulation requires timely suppression of catabolism to prevent excessive hormone accumulation. The cytochrome P450 monooxygenase *CYP707A* catalyzes ABA 8′-hydroxylation, and the resulting 8′-hydroxy-ABA spontaneously isomerizes to the low-activity catabolite phaseic acid (PA) (Kushiro et al., 2004). In grape, *VvCYP707A* maintains relatively high expression before véraison, corresponding to low ABA levels at this stage; following véraison initiation, expression of this gene is markedly downregulated, permitting NCED-driven ABA synthesis to proceed uninhibited by catabolism (Speirs et al., 2013). Exogenous application of the ABA catabolism inhibitor nordihydroguaiaretic acid (NDGA) significantly elevates berry ABA content and accelerates ripening progression, confirming the functional importance of catabolism in ABA homeostasis.

Nevertheless, substantial limitations exist in current grape CYP707A research. Present understanding of VvCYP707A function relies primarily on transcriptional correlations and functional extrapolation from Arabidopsis systems, where CYP707A enzymes have been biochemically characterized and genetically validated through knockout studies (Kushiro et al., 2004). In grape, direct genetic evidence remains lacking. Whether VvCYP707A expression differences among cultivars explain cultivar-specific ABA accumulation patterns remains unverified. Similarly, questions regarding substrate specificity, catalytic efficiency, and tissue localization of this enzyme await experimental validation in grape. Furthermore, ABA-glucose ester (ABA-GE) represents an alternative inactivation pathway whose relative contribution and regulatory mechanisms in grape remain largely unexplored.

### 2.3 The autocatalytic loop and its paradox

A defining feature of ABA homeostasis is its autocatalytic feedback loop, a mechanism that amplifies initial signals and ensures irreversibility of developmental transitions. Exogenous ABA treatment experiments have provided direct evidence: low-concentration ABA application (100–500 μM) to pre-véraison berries not only immediately elevates tissue ABA content but also sustains induction of endogenous *VvNCED* gene expression while simultaneously suppressing *VvCYP707A* transcription, thereby establishing a positive feedback loop wherein “ABA promotes its own synthesis and inhibits its own degradation” (Li et al., 2021). This autocatalytic property explains the “burst” kinetics of ABA accumulation at véraison—once concentration exceeds a threshold, the system becomes self-sustaining and accelerates transition toward a high-ABA state. Proteomic analyses have revealed that ABA treatment can trigger post-translational modifications of key metabolic enzymes even before transcriptional activation occurs (Giribaldi et al., 2010).

This positive feedback system is essential for non-climacteric fruits. Lacking ethylene autocatalysis, grape must rely on ABA self-amplification to ensure unidirectionality of the developmental program: once véraison initiates, even if the initial inducing signal dissipates, the endogenous ABA loop autonomously maintains elevated hormone levels, driving coordinated progression of downstream ripening-related processes (Rattanakon et al., 2016).

However, the autocatalytic model confronts a critical paradox: if the ABA loop possesses intrinsic positive feedback properties, why does this loop fail to initiate or sustain in certain cultivars or developmental conditions? The pink cultivar Benitaka exhibits only moderate ABA accumulation and anthocyanin synthesis even during véraison, suggesting restricted activation of its autocatalytic loop. Does this restriction originate from differences in initial transcriptional activity of the *VvNCED* promoter, or does a “gating” mechanism exist that blocks ABA signal feedback to metabolic genes? The conventional transcription factor regulatory framework cannot fully explain such cultivar specificity, as *VvNCED* promoter sequences are highly conserved across cultivars. This contradictory phenomenon points toward a potentially hidden regulatory layer: epigenetic modifications may determine chromatin states, thereby setting the responsiveness of *VvNCED* and other ABA metabolic genes to developmental or hormonal signals. In other words, ABA may be “poised for burst,” but whether this burst occurs depends on whether chromatin permits transcriptional machinery access to genes. This hypothesis links ABA homeostasis to epigenetic gating, providing a novel entry point for understanding cultivar differences. However, elevated ABA concentration alone cannot explain why cultivars such as Benitaka show attenuated responses despite similar hormonal dynamics—a puzzle that requires examination of downstream signal transduction and, as we will argue, epigenetic gating mechanisms.

## 3 Signal transduction

**FIGURE 2:**
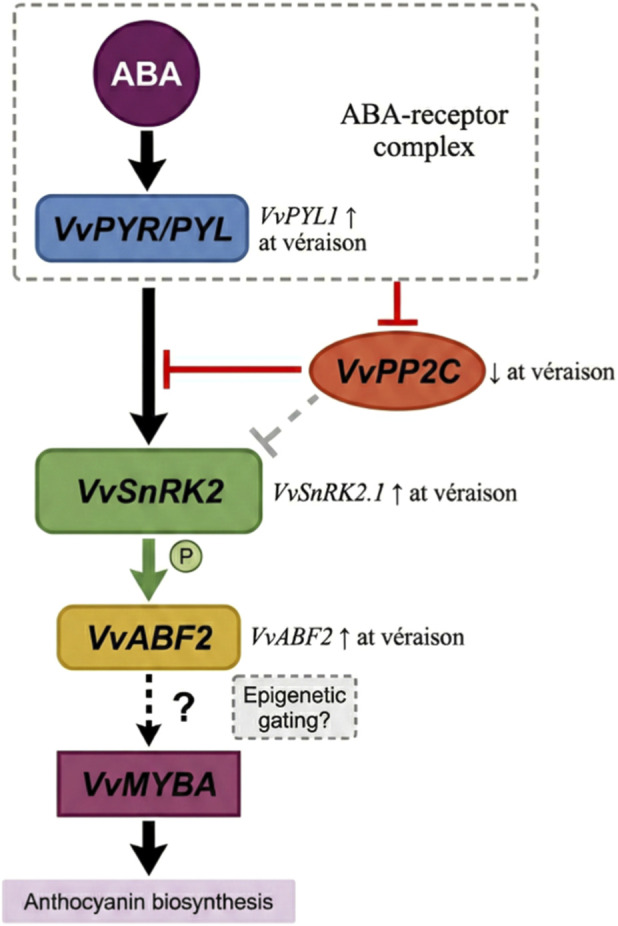
ABA signal transduction cascade model in grape berry.

ABA homeostasis establishes the foundation for hormonal “burst,” but how is this chemical signal perceived and converted into specific transcriptional responses? As illustrated in Figure 2, this conversion relies on a conserved four-tier signaling cascade: ABA is first perceived by *VvPYR/PYL* receptors, which subsequently relieve PP2C-mediated inhibition of SnRK2; activated SnRK2 then phosphorylates and activates *VvABF2*, ultimately driving downstream VvMYBA expression and anthocyanin synthesis. While this module has been systematically characterized in *Arabidopsis*, critical gaps remain in its role in grape berry anthocyanin regulation, particularly regarding the regulatory mechanism between *VvABF2* and VvMYBA.

### 3.1 PYR/PYL receptors as ABA sensors

The PYR/PYL receptor family functions as direct intracellular ABA sensors, converting hormonal signals into protein-protein interaction events through conformational changes. The grape genome encodes 14 PYR/PYL family members (Boneh et al., 2012b). *VvPYL1* is upregulated in berries at véraison, and its heterologous expression partially complements the ABA-insensitive phenotype of *Arabidopsis pyl mutants* (Li et al., 2012). More direct evidence comes from Red Globe grape: VlPYL1 overexpression significantly promotes anthocyanin accumulation in transgenic tobacco, while silencing suppresses berry coloration (Gao et al., 2018). *VvPYL4* is highly expressed in roots and responds to multiple abiotic stresses; its overexpression enhances drought, salt, and freezing tolerance in *Arabidopsis* (Ren et al., 2022), suggesting this receptor may primarily participate in stress responses in vegetative organs. However, which of the 14 PYL members serves as the predominant ABA receptor in berries at véraison remains unclear, and genetic knockout experiments directly demonstrating their necessity are lacking.

### 3.2 PP2C-mediated negative regulation

Type 2C protein phosphatases (PP2Cs) maintain SnRK2 kinases in an inactive state through dephosphorylation. In the absence of ABA, PP2Cs form inhibitory complexes with SnRK2; upon ABA binding to PYR/PYL, the receptor-ABA complex recruits and inhibits PP2Cs, releasing SnRK2 for autophosphorylation and activation (Hubbard et al., 2010). This “double-negative regulation” mechanism (PP2C inhibits SnRK2; ABA-PYR inhibits PP2C) ensures stringent pathway closure under basal conditions and rapid activation upon hormonal stimulation. *VvPP2C3* and *VvPP2C6* are expressed in grape berries (Boneh et al., 2012a). PP2C members maintain high expression before véraison and are downregulated afterward, potentially enhancing ABA sensitivity through reduced negative regulation (Zhang et al., 2021). However, this inference is based on correlational analyses and lacks direct biochemical evidence. Substrate specificity of grape PP2C proteins and their interaction affinities with PYL receptors have not been systematically determined.

### 3.3 SnRK2 as central signal transducer

SNF1-related protein kinase 2 (SnRK2) occupies a critical execution node in the signaling pathway, transmitting hormonal signals through phosphorylation of downstream transcription factors and metabolic enzymes. In Arabidopsis, where this pathway has been most thoroughly characterized, three members—SnRK2.2, SnRK2.3, and SnRK2.6—redundantly regulate ABA responses, with the triple mutant displaying extreme ABA insensitivity (Fujii and Zhu, 2009; Fujita et al., 2009). SnRK2 activation depends on autophosphorylation of specific residues within the activation loop. In grape, VvSnRK2.1 is upregulated at véraison, and its expression highly correlates with ABA accumulation and anthocyanin synthesis (Zhang et al., 2022; Zhang et al., 2021), although functional redundancy among grape SnRK2 members has not been directly tested.

A critical dimension of SnRK2 function is its substrate diversity. Beyond ABF transcription factors, SnRK2 may also phosphorylate epigenetic modifying enzymes. Recent studies have shown that ABA treatment alters DNA methylation patterns of ripening-related genes in grape berries (Li et al., 2024), implying that ABA signaling may regulate epigenetic enzymes through as-yet-unidentified mechanisms. Whether SnRK2 regulates the activity of DNA methyltransferases or demethylases through phosphorylation remains mechanistically plausible but lacks direct evidence. Phosphoproteomic studies may reveal novel substrates, providing a molecular basis for the ABA-epigenetic connection.

### 3.4 ABF-mediated transcriptional response

ABRE-binding factors (ABFs) are bZIP transcription factors that specifically bind ABA-responsive elements, serving as direct SnRK2 substrates that convert kinase activity into transcriptional output. SnRK2-mediated phosphorylation of ABF enhances its transcriptional activation activity (Fujita et al., 2013). *VvABF2* is sharply upregulated at véraison (Nicolas et al., 2014), and its overexpression significantly enhances ABA sensitivity in transgenic *Arabidopsis*. Genome-wide bZIP family analysis has identified multiple ABF homologs expressed in berries (Liu et al., 2014).

However, a puzzling gap exists regarding the direct link between ABF and VvMYBA. Chromatin immunoprecipitation studies have failed to demonstrate direct ABF binding to the VvMYBA promoter. Although the VvMYBA promoter contains putative ABRE elements, their functional significance remains unvalidated. The conventional hypothesis posits that ABF indirectly regulates VvMYBA through intermediate transcription factors, yet this fails to explain why ABA can rapidly induce VvMYBA expression (within hours).

We propose an alternative hypothesis: the primary function of ABF may not be direct activation of VvMYBA transcription but rather “unlocking” chromatin through recruitment of epigenetic modifying enzymes (such as histone acetyltransferases or DNA demethylases), thereby altering chromatin state at the VvMYBA locus and enabling pre-existing MYB transcription factors to bind effectively. If correct, this model would explain the indirect yet essential role of ABF in anthocyanin synthesis, the rapidity of ABA responses, and cultivar differences (wherein initial chromatin state determines whether ABF-mediated “unlocking” is required). We emphasize that this hypothesis currently lacks direct experimental support and represents a priority target for future investigation.

The molecular framework of the ABA signal transduction pathway has been largely established in grape, yet missing critical details weaken the explanatory power of this model. Major knowledge gaps include: the principal functional PYL receptor in berries at véraison remains unidentified; understanding of PP2C function relies almost entirely on extrapolation from the *Arabidopsis* model; the SnRK2 substrate spectrum is incomplete; and direct ABF-VvMYBA interaction has not been confirmed. These gaps collectively point to a core question: can the established linear model (ABA → PYR/PP2C/SnRK2 → ABF → VvMYBA) fully explain observed biological phenomena? Complexities such as cultivar differences, environmental memory, and signal specificity suggest that an active “decision layer”—an epigenetic gating mechanism—may exist between classical signal transduction and transcriptional activation. The ABA signal, like a “messenger,” has already arrived, but whether it can be “delivered” to the VvMYBA gene may depend not only on the messenger itself but also on whether the “gate” at the gene locus is open. The failure to detect direct ABF binding to the VvMYBA promoter, together with marked cultivar differences in ABA responsiveness, suggests that an intermediate regulatory layer—epigenetic modifications—may gate the accessibility of this locus to transcription factors.

## 4 Epigenetic gating: the decision layer

**FIGURE 3:**
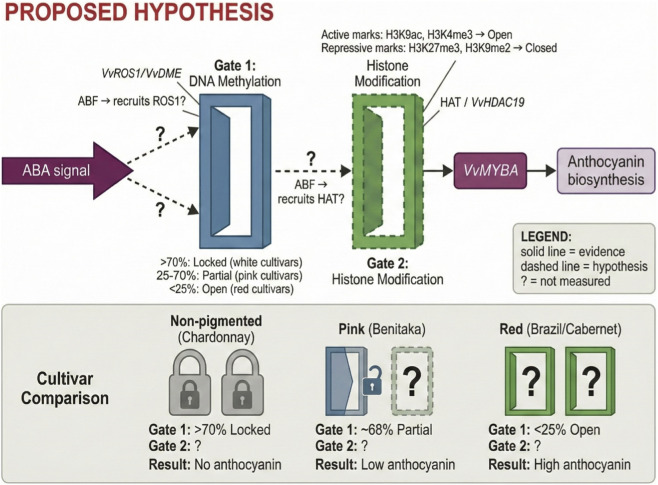
The proposed epigenetic dual-gating hypothesis. Notes: Solid arrows indicate evidence-supported connections; dashed arrows indicate hypothesized connections; question marks (?) denote parameters not yet measured in grape. Gate 1 status is based on bisulfite sequencing data from grape cultivars; Gate 2 status is inferred from Arabidopsis studies.

The preceding sections established the molecular framework from ABA perception to signal transduction, yet a core contradiction remains unresolved: why do similar ABA signals produce markedly different transcriptional responses across cultivars or environmental conditions? This chapter proposes a working hypothesis for a novel regulatory tier—the epigenetic gating mechanism—that may actively determine whether ABA signals can be converted into transcriptional output. We note at the outset that while correlative evidence supports this model (particularly for Gate 1), the mechanistic connections between ABA signaling and epigenetic enzyme recruitment remain to be experimentally validated. As illustrated in Figure 3, this “decision layer” is proposed to comprise a “dual-gating” system consisting of DNA methylation (Gate 1) and histone modifications (Gate 2); ABA signals must sequentially pass through both gates to ultimately activate VvMYBA expression and anthocyanin synthesis. This conceptual framework aims to provide a unified explanation for cultivar specificity and environmental plasticity of anthocyanin synthesis.

### 4.1 The necessity of chromatin-based gating

The necessity of epigenetic regulation as an independent regulatory layer stems from three empirical phenomena that cannot be explained by genetic or signaling pathway differences alone. The cultivars Brazil and Benitaka share identical VvMYBA coding sequences, both lacking Gret1 insertion, yet Brazil displays deep red coloration while Benitaka appears only pink. Whole-genome bisulfite sequencing revealed a critical distinction: Benitaka maintains 68% DNA methylation in the VvMYBA regulatory region (retrotransposon 3′LTR), whereas Brazil shows only 22% (Azuma and Kobayashi, 2022). This methylation difference mirrors gene expression patterns—VvMYBA expression in Brazil significantly exceeds that in Benitaka. Injection of the DNA methylation inhibitor 5-azacytidine restores red phenotypes in non-pigmented grape berries (Azuma et al., 2009), directly demonstrating that methylation, rather than DNA sequence, is the determinant of pigment synthesis.

Extreme inter-cultivar differences further support a chromatin state “locking” mechanism (Figure 3, lower panel cultivar comparison). In non-pigmented cultivars such as Chardonnay, DNA methylation levels at the VvMYBA locus exceed 70%, with both gates locked, resulting in complete silencing (Shangguan et al., 2020). This “permanent lockdown” cannot be relieved by exogenous ABA treatment. In contrast, red cultivars such as Cabernet Sauvignon and Brazil maintain low methylation (<25%), preserving high gene responsiveness to developmental or hormonal signals (Jia et al., 2020). Pink cultivars occupy an intermediate state, with their partial methylation (40%–60%) potentially explaining sensitivity to environmental stimuli.

Environmental memory phenomena reveal the temporal dimension of epigenetic regulation. Heat stress not only suppresses current anthocyanin synthesis but also establishes long-term repressive states through histone modifications. Studies in *Arabidopsis* have demonstrated that heat stress rapidly induces H3K27me3 deposition at stress-responsive gene loci, with this repressive mark persisting for weeks (Nishio et al., 2024). Sustained suppression of anthocyanin synthesis following high-temperature treatment in grape berries suggests similar mechanisms may exist (Mori et al., 2007). The histone deacetylase VvHDAC19 establishes a repressive chromatin state at the VvMYB5a locus by recruiting the transcriptional repressor ERF4 (Jia et al., 2023), further supporting the notion that epigenetic modifications integrate historical information to shape future responses.

### 4.2 DNA methylation: the primary gate

DNA methylation serves as the “first gate,” directly impeding transcription factor binding or recruiting methyl-binding protein-mediated transcriptional repression through methyl group addition in CG, CHG, and CHH sequence contexts. Genome-wide DNA methylation profiling during grape berry development has revealed its dynamic and locus-specific nature. Véraison is accompanied by genome-wide methylation remodeling, with ripening-related genes tending toward demethylation to facilitate expression (Tang et al., 2020). CHH context hypermethylation accumulation in transposons and genic regions correlates with precocious phenotypes.

Establishment and maintenance of DNA methylation depend on conserved methyltransferase families. The grape genome encodes VvMET1 for CG methylation maintenance and VvDRM2 for *de novo* methylation (Lucibelli et al., 2022). DNA demethylation occurs through active processes catalyzed by DNA glycosylases including VvROS1 and VvDME, although specific functions of these enzymes in berry development have not been directly characterized through genetic studies. Studies of tomato SlDML2 provide important cross-species reference: this gene is specifically upregulated at ripening initiation, and its mutation causes hypermethylation and expression suppression of ripening-related genes (Liu et al., 2015; Lang et al., 2017), establishing the critical role of active demethylation in climacteric fruit ripening. By analogy, grape demethylases may function similarly during véraison, though this awaits direct experimental confirmation given the distinct ripening physiology of non-climacteric fruits.

How ABA regulates DNA methylation enzymes constitutes the most critical yet weakest link between signal transduction and epigenetic modification—and represents the central uncertainty of our hypothesis. Recent studies have shown that ABA treatment significantly alters methylation patterns of ripening-related genes in grape berries (Li et al., 2024), suggesting that ABA signaling pathways may regulate methylation enzyme function. However, a substantial gap exists between this observation and the underlying molecular mechanisms. Does ABF transcription factor directly recruit VvROS1 to the VvMYBA promoter? Does SnRK2 kinase regulate methylation enzyme activity or localization through phosphorylation? These critical questions entirely lack experimental evidence, and we acknowledge that our model’s central mechanistic claims rest on inference rather than demonstrated molecular interactions.

### 4.3 Histone modifications: the secondary gate

Even when DNA methylation permits transcription factor access to genes, higher-order chromatin structure may still impede transcriptional machinery assembly. Histone modifications serve as the “second gate,” regulating gene transcriptional activity by altering nucleosome-DNA binding affinity or recruiting chromatin remodeling factors. Activating modifications such as H3K9ac and H3K4me3 mark open chromatin and active transcription; repressive modifications such as H3K9me2/3 and H3K27me3 are associated with heterochromatin and gene silencing (Li et al., 2022). Studies of strawberry fruit ripening have revealed dynamic histone modification remodeling: ripening-related genes acquire H3K4me3 and H3K9ac marks at véraison, while early developmental genes are suppressed by H3K27me3 marking (Pan et al., 2024).

Histone deacetylases (HDACs) promote chromatin compaction by removing acetyl groups, while histone acetyltransferases (HATs) catalyze the reverse process. *VvHDAC19* interacts with the *VvMYB5a* promoter by recruiting the transcriptional repressor ERF4, establishing a deacetylated repressive chromatin state that reduces anthocyanin accumulation (Jia et al., 2023). Silencing of *VvHDAC19* significantly enhances anthocyanin synthesis, confirming the negative regulatory role of HDACs. Systematic genome-wide analysis of lysine acetyltransferase and deacetylase families has revealed their differential expression in grape abiotic stress responses, but functions of specific HAT members in berry ripening and anthocyanin synthesis remain unidentified. Ripening-associated histone modification dynamics have been characterized in strawberry (Pan et al., 2024), and single-base resolution methylome profiling has revealed dramatic epigenetic remodeling during tomato fruit development (Zhong et al., 2013), suggesting that chromatin-level regulation is a conserved feature of fleshy fruit ripening. Antagonistic interactions between the H2A.Z variant and H3K4me3 regulate anthocyanin biosynthesis genes in *Arabidopsis* (Cai et al., 2019), suggesting coordinated action between chromatin variants and histone modifications.

Heat stress rapidly induces H3K27me3 deposition at stress-responsive genes in Arabidopsis, with this modification persisting for weeks and conferring “pre-adaptation” to subsequent stress (Nishio et al., 2024). This memory depends on H3K27me3 catalyzed by Polycomb Repressive Complex 2 (PRC2). It must be emphasized that this mechanism has only been validated in Arabidopsis; PRC2 function in grape berry development and stress responses has not been directly investigated. While sustained suppression of anthocyanin synthesis following high-temperature treatment (Mori et al., 2007; Gouot et al., 2019) is consistent with similar mechanisms operating in grape, this interpretation remains speculative. Critically, histone modification states at the VvMYBA locus under different temperature treatments have not been directly measured by ChIP-qPCR or ChIP-seq, representing one of the most important gaps that must be addressed to validate the epigenetic memory component of our hypothesis.

How ABA signaling regulates histone-modifying enzymes represents the weakest link in the epigenetic gating mechanism. One hypothesis proposes that ABF transcription factors, upon phosphorylation activation by SnRK2, acquire the ability to recruit chromatin-modifying enzymes such as HATs, guiding them to the VvMYBA locus to catalyze histone acetylation and open chromatin. However, this mechanistic model is entirely speculative. Key questions include: Do ABF and HAT physically interact? Does this interaction depend on ABF phosphorylation status? Do histone acetylation levels at the VvMYBA locus increase following ABA treatment? Addressing these questions requires Co-IP validation of ABF-HAT interactions, ChIP-qPCR detection of ABA-responsive acetylation levels at the VvMYBA locus, and ChIP-reChIP demonstrating co-localization of ABF and HAT on chromatin. Until these experiments are completed, “ABF recruits HAT” should be explicitly identified as a testable but unvalidated working hypothesis.

Chromatin remodeling complexes such as SWI/SNF alter DNA accessibility by sliding or evicting nucleosomes (Guo et al., 2022). In *Arabidopsis*, SWI/SNF complexes participate in precise regulation of developmental transitions and stress responses, but their function in grape berries remains entirely unknown.

### 4.4 The dual-gating model

Integrating correlative evidence from DNA methylation studies with inferences from histone modification research, this review proposes a “dual-gating” working hypothesis that aims to provide a unified conceptual framework for the complexity of anthocyanin regulation. As illustrated in Figure 3, VvMYBA gene expression is proposed to depend on two sequential epigenetic “gates”: the first gate (DNA methylation) exists in three states based on methylation level—locked (>70%, non-pigmented cultivars), partially open (25%–70%, pink cultivars), and fully open (<25%, red cultivars); the second gate (histone modifications) is hypothesized to be determined by the balance between activating marks (H3K9ac, H3K4me3) and repressive marks (H3K27me3, H3K9me2). According to this model, both gates must be simultaneously open for high-level gene expression. Importantly, Gate 1 is supported by direct bisulfite sequencing data from grape cultivars (Azuma and Kobayashi, 2022; Shangguan et al., 2020), whereas Gate 2 status is inferred from Arabidopsis studies and has not been directly characterized at the VvMYBA locus.

If validated, this model would provide unified explanations for the three phenomena raised at the beginning of this chapter. According to our hypothesis, cultivar differences may originate from genetically fixed states of the first gate: VvMYBA promoters in non-pigmented cultivars are permanently “locked” by hypermethylation; even with sufficient ABA signaling and normal histone modifications, transcription factors cannot access the gene. The Brazil *versus* Benitaka contrast refines this mechanism: Brazil’s low methylation (22%) means the first gate is fully open, allowing ABA signals to act directly on the second gate; Benitaka’s intermediate methylation (68%) forms a partial barrier, requiring stronger demethylation activity or higher ABA concentrations for full gene activation. Environmental memory operates primarily through the second gate: heat stress-induced H3K27me3 deposition closes the second gate, and this modification can persist for weeks even after temperature recovery, preventing anthocyanin synthesis restoration. This memory is independent of DNA methylation and can therefore be established even in low-methylation cultivars. Signal specificity is reflected in differential ABA regulation of both gates: acting on the first gate through demethylase recruitment (hypothesized) and on the second gate through HAT recruitment (hypothesized). Initial states of both gates in different cultivars or developmental stages determine ABA signal effectiveness—if either gate is locked, no amount of signal input can produce transcriptional output.

The significance of this model lies in redefining the epigenetic layer from a passive “recorder” to an active “decision-maker.” In traditional models, epigenetic modifications were viewed as consequences of transcriptional states; the dual-gating model instead confers causality and regulatory function—epigenetic states determine whether genes can respond to signals. This paradigm shift has profound implications for understanding developmental plasticity, cultivar breeding, and environmental adaptation. However, it must be emphasized that the core links of this model—how ABA regulates both gates—remain primarily speculative rather than directly evidenced. Transforming this conceptual framework into mechanistic understanding requires extensive systematic experimental validation. When both gates are open—as proposed to occur in deeply colored cultivars—the transcriptional machinery can engage with the VvMYBA promoter, activating the downstream regulatory network described in the following section.

## 5 Transcriptional network

**FIGURE 4:**
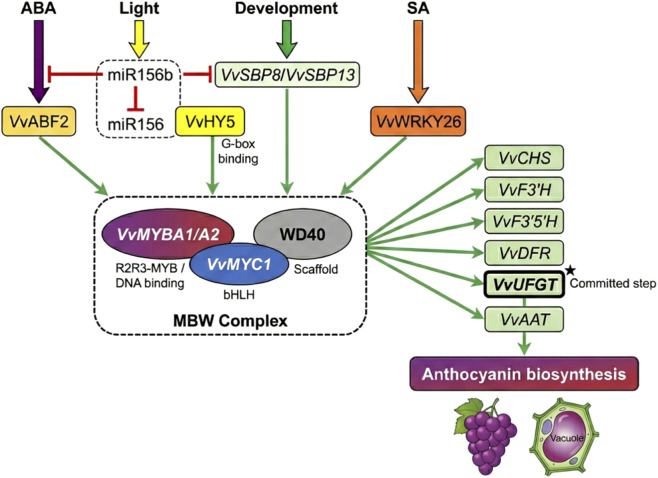
Transcriptional regulatory network of anthocyanin synthesis in grape berry.

The “unlocking” of epigenetic gates creates conditions for transcription factor access to the VvMYBA gene, but ultimate gene activation depends on a complex transcriptional regulatory network. As illustrated in Figure 4, this network integrates inputs from multiple signals including ABA, light, developmental programs, and salicylic acid, coordinating activation of anthocyanin biosynthesis structural genes through the MYB-bHLH-WD40 (MBW) ternary complex.

### 5.1 Upstream transcriptional regulators

As the master switch of anthocyanin regulation, VvMYBA expression is coordinately regulated by multiple upstream transcription factors. Beyond the aforementioned ABA response factor ABF, *VvHY5* (ELONGATED HYPOCOTYL 5)—a core regulator of light signaling—integrates light signals with anthocyanin synthesis by binding G-box elements in the promoter (Carbonell-Bejerano et al., 2014). Small RNA-mediated post-transcriptional regulation adds an additional regulatory dimension to the network. This miRNA-mediated regulation involves complex hierarchical relationships. ABA downregulates *miR156b* expression, and mutual regulation exists between *miR156b* and *miR156;* miR156 suppresses SPL/SBP transcription factors including *VvSBP8* and *VvSBP13* through targeting (Figure 4). ABA signaling ultimately relieves repression of *VvSBP8* and *VvSBP13*, which subsequently activate anthocyanin biosynthesis, revealing deep integration between developmental programs and metabolic regulation. The WRKY transcription factor family also participates in this regulation: *VvWRKY26* acts synergistically with the MBW complex, is induced by salicylic acid, and promotes anthocyanin accumulation (Zhang et al., 2025).

### 5.2 The MBW transcriptional complex

The MYB-bHLH-WD40 (MBW) ternary complex constitutes the core execution unit of anthocyanin biosynthesis transcriptional regulation. As shown in Figure 4, the grape MBW complex comprises R2R3-MYB transcription factors (*VvMYBA1/VvMYBA2*, responsible for DNA binding), bHLH transcription factors (V*vMYC1*), and WD40 repeat proteins (serving as scaffolds). VvMYBA proteins provide DNA-binding specificity, recognizing MYB recognition elements in structural gene promoters; *VvMYC1* enhances complex stability and transcriptional activation activity through physical interaction with VvMYBA (Hichri et al., 2010); WD40 proteins serve as scaffolds to facilitate MYB-bHLH interactions.

Functional differentiation and allelic variation within the VvMYBA gene family constitute the genetic basis for cultivar color diversity. *VvMYBA2* exists as functional allele *VvMYBA2r* (red allele) and non-functional allele *VvMYBA2w*, with the latter losing expression due to promoter deletion (Jiu et al., 2021). Different VvMYBA allele combinations determine cultivar pigmentation capacity and patterns. Other MYB family members such as VvMYB86 antagonistically regulate proanthocyanidin and anthocyanin synthesis, achieving metabolic flux redistribution through competitive binding to bHLH partners or target gene promoters (Cheng et al., 2021).

### 5.3 Downstream target genes

The MBW complex coordinates the entire anthocyanin biosynthesis pathway by activating multiple structural genes. UDP-glucose:flavonoid 3-O-glucosyltransferase (*VvUFGT*), as a key target gene, encodes the enzyme catalyzing the committed step of anthocyanin biosynthesis; its promoter contains multiple MRE and bHLH binding sites. *VvUFGT* expression exhibits strict spatiotemporal correlation with anthocyanin accumulation. As illustrated in Figure 4, the MBW complex coordinately activates structural genes throughout the pathway, including *VvCHS*, *VvF3′H, VvF3′5′H*, *VvDFR*, *VvUFGT*, and *VvAAT*, ensuring unimpeded metabolic flux from primary metabolites to vacuolar storage (Rinaldo et al., 2015). This coordinated activation ensures smooth metabolic flow from primary metabolites to end products. However, transcript abundance alone does not guarantee metabolic output—the biosynthetic enzymes must execute the program, with VvUFGT-catalyzed glycosylation representing the committed step.

## 6 Metabolic pathway

**FIGURE 5:**
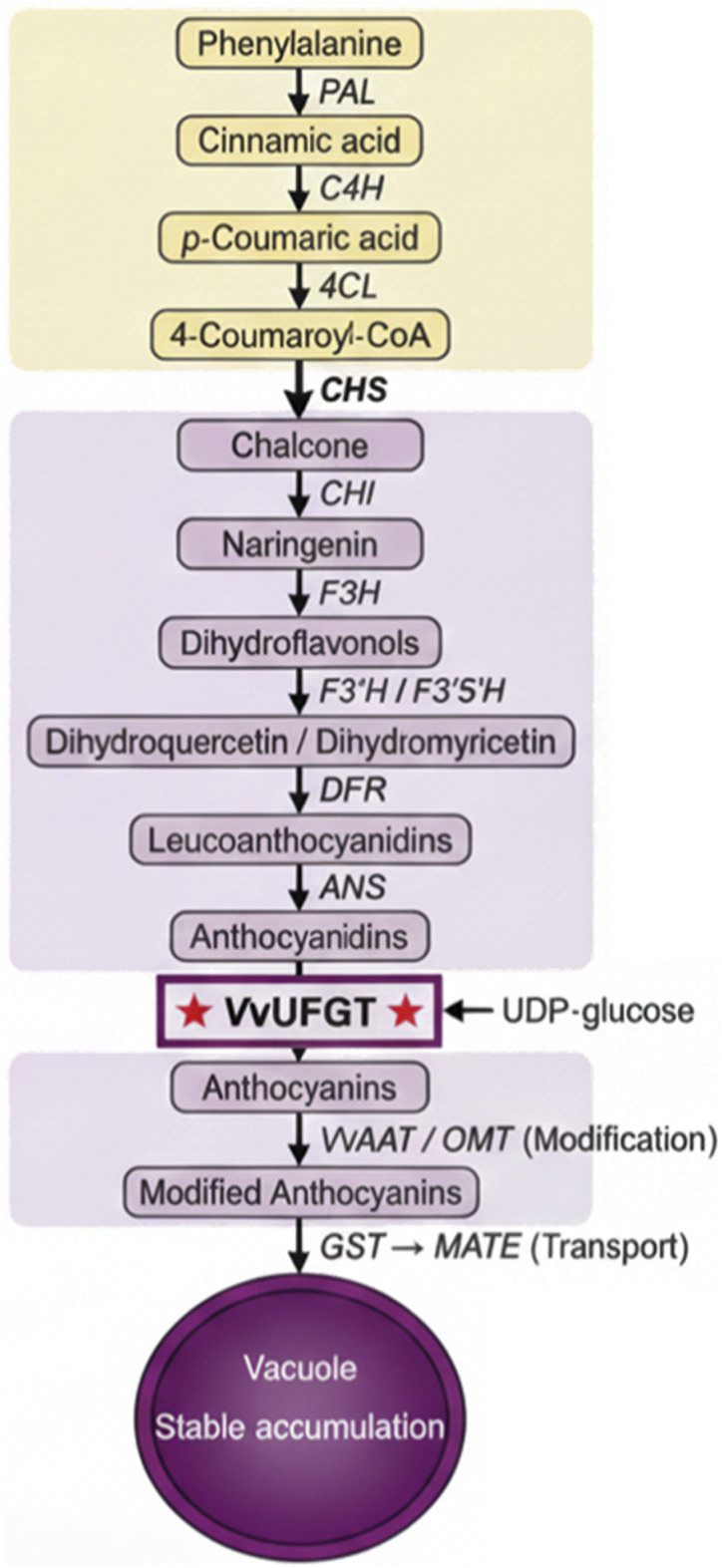
Anthocyanin biosynthesis metabolic pathway in grape berry.

The structural genes activated by the transcriptional network cooperatively catalyze the complex biochemical conversion from phenylalanine to anthocyanins. As illustrated in Figure 5, this metabolic pathway comprises three stages: the general phenylpropanoid pathway, the flavonoid branch pathway, and anthocyanin-specific synthesis and modification, ultimately ensuring stable pigment accumulation in vacuoles through transport systems.

### 6.1 The phenylpropanoid pathway

Anthocyanin biosynthesis initiates from the phenylpropanoid metabolic pathway, which uses phenylalanine as substrate and converts it to 4-coumaroyl-CoA through phenylalanine ammonia-lyase (PAL), cinnamate 4-hydroxylase (C4H), and 4-coumarate:CoA ligase (4CL). This general pathway provides precursors for various secondary metabolites including flavonoids, lignins, and stilbenes. In the flavonoid branch, chalcone synthase (CHS) catalyzes chalcone formation, which is isomerized by chalcone isomerase (CHI) to naringenin, subsequently forming dihydroflavonols through catalysis by flavonoid 3-hydroxylase (F3H), flavonoid 3′-hydroxylase (F3′H), and flavonoid 3′,5′-hydroxylase (F3′5′H) (Shi et al., 2024). Dihydroflavonol reductase (DFR) reduces dihydroflavonols to leucoanthocyanidins, and anthocyanidin synthase (ANS/LDOX) subsequently catalyzes oxidation of leucoanthocyanidins to colored anthocyanidins. These two reactions determine the metabolic partitioning between anthocyanins and other flavonoids (Yang et al., 2022).

### 6.2 VvUFGT: the committed step

Anthocyanidins are unstable and exhibit low biological activity, requiring glycosylation modification for stable accumulation. UDP-glucose:flavonoid 3-O-glucosyltransferase (UFGT) catalyzes transfer of a glucose moiety from UDP-glucose to the 3-position hydroxyl group of anthocyanidins, generating stable anthocyanin monoglucosides. As shown in Figure 5, this step represents the committed step of anthocyanin biosynthesis. The grape *VvUFGT* gene is sharply upregulated at véraison, with its expression pattern highly consistent with anthocyanin accumulation. *VvUFGT* exhibits differential catalytic efficiency toward different anthocyanidins, preferentially catalyzing glycosylation of cyanidin and delphinidin. Grape anthocyanins exist almost exclusively as monoglucosides, reflecting the unique biochemical properties of grape UFGT. *VvUFGT* transcriptional regulation is directly controlled by the MBW complex, with its promoter being a canonical target of VvMYBA.

### 6.3 Modification and transport

Anthocyanin monoglucosides require further modification and transport for stable vacuolar accumulation. Anthocyanin acyltransferases (e.g., *VvAAT*) catalyze transfer of acyl groups such as acetyl or coumaroyl moieties, enhancing anthocyanin stability and color intensity (Rinaldo et al., 2015). O-methyltransferases (OMTs) catalyze methylation of B-ring hydroxyl groups, altering pigment hue and stability. Vacuolar transport of anthocyanins represents the final critical step in pigment accumulation. Glutathione S-transferases (GSTs) function as anthocyanin-binding proteins, transporting anthocyanins from synthesis sites to vacuoles via vesicle-mediated pathways (Conn et al., 2008). MATE (multidrug and toxic compound extrusion) family transporters on the tonoplast serve as H^+^ gradient-driven secondary active transporters, mediating anthocyanin translocation across the vacuolar membrane (Gomez et al., 2009). Transport efficiency directly influences final anthocyanin accumulation levels. This entire regulatory cascade—from ABA homeostasis through epigenetic gating to metabolic execution—operates within dynamic environmental contexts that continuously modulate pathway activity.

## 7 Environmental integration

**FIGURE 6:**
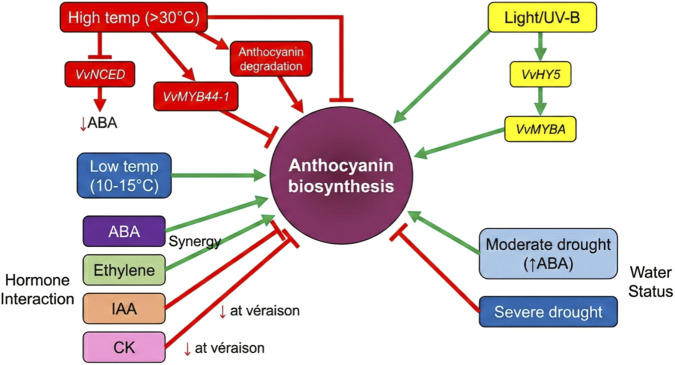
Integrated regulation of anthocyanin synthesis in grape berry by environmental factors.

The complex regulatory network of anthocyanin biosynthesis enables responses to diverse environmental signals. As illustrated in Figure 6, environmental factors including temperature, light, water status, and hormonal crosstalk collectively regulate anthocyanin synthesis, with promoting factors (low temperature, light/UV-B, moderate drought, ABA, and ethylene) and inhibiting factors (high temperature, severe drought, IAA, and cytokinin) forming a dynamic equilibrium.

### 7.1 Temperature: multilayered regulation and epigenetic memory

Temperature represents the most important environmental factor affecting grape anthocyanin accumulation. High temperatures (>30 °C–35 °C) significantly inhibit anthocyanin synthesis during véraison through multiple levels. At the ABA level, high temperature suppresses *VvNCED* expression while promoting *VvCYP707A* activity, reducing endogenous ABA levels (Yamane et al., 2006). At the transcriptional level, high temperature inhibits anthocyanin biosynthesis through the VvFHY3-ARF3 module: *VvFHY3*, a light signaling factor, is downregulated under high temperature, relieving repression of the auxin response factor ARF3, which negatively regulates anthocyanin synthesis through modulation of endoplasmic reticulum stress responses (Sun et al., 2025). *VvMYB44-1*, a heat-responsive transcription factor, is upregulated under high temperature and directly inhibits anthocyanin biosynthesis gene expression. At the metabolic level, high temperature enhances peroxidase activity, promotes degradation of synthesized anthocyanins (Movahed et al., 2016).

High temperature-mediated suppression of anthocyanin synthesis exhibits persistence; even after temperature recovery, pigment accumulation restoration is significantly delayed (Ryu et al., 2020). The molecular basis of this “environmental memory” likely involves epigenetic modifications. In *Arabidopsis*, heat stress induces H3K27me3 deposition at stress-responsive gene loci, with this modification persisting for weeks. Low temperature, conversely, promotes anthocyanin synthesis. Nocturnal low temperatures (10 °C–15 °C) during véraison significantly enhance grape anthocyanin accumulation without corresponding increases in ABA levels (Gao-Takai et al., 2019), suggesting that low temperature may act through ABA-independent pathways. Inter-cultivar differences in temperature response constitute an important component of climate adaptability.

### 7.2 Hormonal crosstalk

Although ABA is the central hormone in anthocyanin regulation, its action is modulated and integrated by other hormones. Auxin (IAA) forms an antagonistic relationship with ABA: high IAA levels inhibit ripening initiation and anthocyanin synthesis, and declining IAA content at véraison is a prerequisite for ripening initiation (Ziliotto et al., 2012). Ethylene, while not the primary driver of non-climacteric fruit ripening, synergistically promotes anthocyanin synthesis with ABA (Böttcher et al., 2013). Exogenous ethylene or ethylene precursor treatment accelerates véraison, whereas ethylene biosynthesis or signaling inhibitors delay ripening. Cytokinins (CKs), as growth-promoting hormones, inhibit ripening and anthocyanin synthesis at high levels. CK levels decline at véraison, and changes in CK signaling pathway gene expression correlate with ripening initiation. These hormonal interactions form an intricate regulatory network at véraison: declining IAA and CK relieve repression of ripening, rising ABA actively promotes ripening, and ethylene synergy enhances ABA signaling.

### 7.3 Light and water availability

Light promotes anthocyanin synthesis through multiple mechanisms. UV-B radiation upregulates VvMYBA and structural gene expression by activating UV-B-specific signaling pathways (Carbonell-Bejerano et al., 2014). Light signals converge with ABA signaling at the VvMYBA promoter through transcription factors such as *VvHY5,* forming synergistic activation. Water status indirectly regulates anthocyanin synthesis by affecting ABA levels and carbohydrate supply. Moderate drought stress enhances ABA accumulation and promotes anthocyanin synthesis (Wang et al., 2022). However, severe water deficit inhibits overall metabolism and reduces anthocyanin content. Sugars, functioning as both signaling molecules and metabolic precursors, play critical roles in the water-ABA-anthocyanin relationship.

## 8 Discussion

**FIGURE 7:**
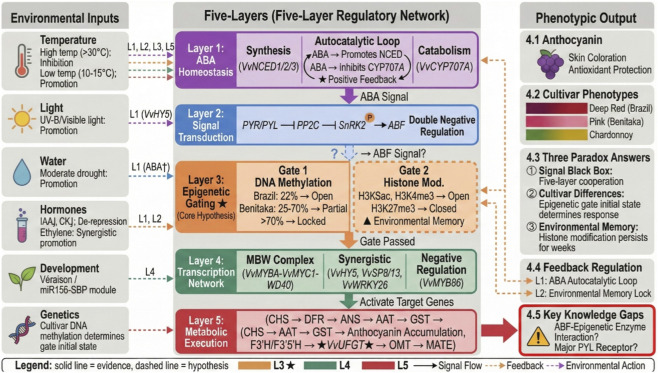
Integrative framework of the five-layer regulatory network for anthocyanin biosynthesis in grape berry. Notes: Layer 3 (epigenetic gating) represents the proposed dual-gating hypothesis; other layers are based on published experimental evidence.

The preceding sections have systematically dissected each tier of grape anthocyanin biosynthesis regulation—from ABA metabolic homeostasis to signaling cascades, from epigenetic modifications to transcriptional network activation, and onward to metabolic pathway execution and environmental factor integration. However, these tiers do not operate as independent modules but rather as nested, dynamically interacting regulatory networks. This review proposes a “five-layer symphony” integrative framework. As illustrated in Figure 7, this framework organizes the regulatory network into five functional layers (ABA homeostasis → signal transduction → epigenetic gating → transcriptional network → metabolic execution), integrating multiple environmental inputs (temperature, light, water, hormones, development, and genetics) to generate phenotypic outputs. Notably, the epigenetic layer is repositioned as an active decision-making center, providing unified explanations for cultivar differences, environmental memory, and signal specificity. Anthocyanin biosynthesis regulation can be abstracted as the coordinated operation of five functional layers.

Layer 1 (ABA homeostasis) establishes hormonal “burst” through precise balance between *VvNCED* and *VvCYP707A*, with its autocatalytic loop ensuring unidirectionality of véraison signaling. However, a critical paradox remains unresolved: why does the autocatalytic loop fail to initiate in Benitaka? Pre-véraison ABA levels are similar in both cultivars, yet Brazil rapidly enters autocatalysis while Benitaka lags behind. This phenomenon cannot be explained by differences in ABA metabolic enzymes, as *VvNCED* and *VvCYP707A* sequences are highly conserved between cultivars. The existence of this paradox suggests that loop initiation requires permission from upper regulatory layers. Layer 2 (signal transduction) converts ABA chemical signals into protein phosphorylation signals through the PYR/PYL-PP2C-SnRK2-ABF cascade. The “double-negative regulation” design of this layer ensures stringent on-off characteristics of the signaling pathway. However, a puzzling gap exists in the direct connection between ABF and VvMYBA: chromatin immunoprecipitation studies have failed to demonstrate direct ABF binding to the VvMYBA promoter, and ABA treatment induces VvMYBA expression within hours—a speed difficult to explain by classical transcription factor cascades. These observations collectively point toward a hidden regulatory layer. Layer 3 (epigenetic gating) represents the core innovation of this review. Traditional models view epigenetic modifications as passive recorders of transcriptional states, whereas the “dual-gating” model proposed here assigns them an active decision-making function: DNA methylation constitutes the “first gate,” determining whether transcription factors can physically access the gene; histone modifications constitute the “second gate,” determining whether chromatin exists in a transcriptionally permissive state. As shown in Figure 7, both gates must be simultaneously open (Gating Logic: Gate 1 AND Gate 2) for VvMYBA to respond to ABA signals; closure of either gate blocks signal transmission.

This model provides unified explanations for three core phenomena. Cultivar differences originate from genetically fixed states of the first gate: VvMYBA promoters in non-pigmented cultivars are permanently “locked” by hypermethylation (>70%), precluding activation even with sufficient ABA; Brazil’s low methylation (22%) leaves the first gate fully open, whereas Benitaka’s intermediate methylation (68%) forms a partial barrier, explaining why the latter requires stronger ABA signals or longer duration to initiate the autocatalytic loop. Environmental memory operates primarily through the second gate: heat stress induces H3K27me3 deposition, with this repressive mark persisting for weeks and preventing anthocyanin synthesis even after temperature recovery. Signal specificity may be reflected in differential ABA regulation of both gates: according to the hypothesis proposed here, ABA may act on the first gate by recruiting demethylases and on the second gate by recruiting histone acetyltransferases (this mechanism awaits experimental validation, indicated by dashed lines in Figure 7). Initial states of both gates in different cultivars or developmental stages determine ABA signal effectiveness. Layer 4 (transcriptional network) activates structural genes after epigenetic “unlocking.” The MBW complex serves as the core execution unit, integrating multiple inputs from ABF (ABA signaling), *VvHY5* (light signaling), and *VvSBP* (developmental signaling) to coordinately activate key target genes including VvUFGT. Discovery of the *miR156-VvSBP* module reveals direct connections between ABA signaling and developmental timing programs, suggesting that anthocyanin synthesis initiation requires not only hormonal signals but also developmental program permission. Layer 5 (metabolic execution) converts transcriptional instructions into biochemical products. *VvUFGT*-catalyzed glycosylation is the committed step, with subsequent acylation, methylation, and vacuolar transport ensuring stable pigment accumulation. Regulation at this layer is relatively autonomous, yet UDP-glucose supply depends on primary metabolism, revealing deep connections between secondary metabolism and development.

Interactions among the five layers constitute the “symphony” character of regulation. The ABA homeostasis layer provides input to the signal transduction layer, but whether signals can be converted to transcriptional output depends on the “gating” status of the epigenetic layer. The epigenetic layer not only responds to current signals but also integrates historical information (environmental memory) and genetic programs (cultivar differences), achieving spatiotemporal-specific regulation. The transcriptional network layer activates the metabolic execution layer under epigenetic permission, while metabolic products (such as anthocyanins) may influence upstream regulation through feedback mechanisms. As shown on the left side of Figure 7, environmental factors exert influence at multiple layers: temperature acts on L1, L2, L3, and L5; light acts on L4 through *VvHY5*; water acts on L1 through ABA; hormonal crosstalk affects L1 and L2; developmental programs act on L4 through the miR156-SBP module; and genetic background determines the initial state of L3 through DNA methylation. Hormonal crosstalk converges mainly at the signaling and transcriptional layers: declining auxin and cytokinin relieve repression of ripening, while ethylene synergistically enhances ABA signaling.

A fundamental question concerns the extent to which regulatory mechanisms characterized in Arabidopsis and tomato are conserved in grape (Table 1). The core ABA signaling module (PYR/PYL-PP2C-SnRK2-ABF) shows functional conservation based on complementation studies, and the general logic that DNA methylation represses gene expression appears universal. However, several aspects may diverge: the specific transcription factors mediating ABA-epigenetic connections, the relative importance of different methylation contexts, and critically, the ripening regulatory logic—grape is non-climacteric while tomato relies on ethylene-mediated climacteric ripening. Grape-specific features, including the unique developmental transition of véraison, the presence of Gret1 retrotransposon insertions, and perennial growth habit, may influence how conserved mechanisms operate in this species. These considerations underscore the need for direct experimental validation in grape rather than uncritical extrapolation from model systems.

**TABLE 1:** Evidence status for key ABA-epigenetic regulatory mechanisms across species.

Mechanism	Arabidopsis	Tomato	Grape	Conservation
PYR/PYL-PP2C-SnRK2	Genetic validation	Partial	*VvPYL1* complementation	Likely conserved
ABF phosphorylation	Biochemical evidence	Partial	Expression correlation only	Presumed conserved
DNA demethylation in ripening	Limited fruit data	*SlDML2* knockout validated	Pharmacological (5-aza)	Hypothesized in grape
H3K27me3 heat memory	ChIP validated	Not tested	Phenotype inference only	Speculative in grape
HDAC regulation	Limited	Limited	*VvHDAC19* functional study	Direct grape evidence
SWI/SNF remodeling	Characterized	Limited	Not studied	Unknown in grape

Evidence categories are defined as follows: Likely conserved, functional complementation or biochemical validation exists in grape; Presumed conserved, expression correlation data support conservation, but direct functional evidence is lacking; Hypothesized in grape, indirect experimental support exists (e.g., pharmacological treatment, phenotypic correlation); Speculative in grape, inferred solely from other species, no experimental data in grape; Unknown, not yet investigated in any species relevant to this pathway.

However, limitations of this framework must be acknowledged. The most critical weakness lies in the completely unknown molecular connection between ABA signaling and epigenetic modifications. The hypotheses proposed here—SnRK2 phosphorylation of epigenetic enzymes, ABF recruitment of demethylases and histone acetyltransferases—are mechanistically plausible but entirely lack direct evidence. Supporting these hypotheses requires multiple experiments: (1) Co-IP validation of direct ABF-ROS1, ABF-HAT, and SnRK2-ROS1 interactions; (2) ChIP-seq identification of whether ROS1 and HAT specifically enrich at the VvMYBA locus following ABA treatment; (3) *in vitro* kinase assays verifying whether SnRK2 can phosphorylate ROS1 or HAT and alter their activity; (4) ChIP-qPCR detection of dynamic changes in methylation and histone acetylation levels at the VvMYBA locus following ABA treatment; (5) genetic experiments using CRISPR knockout to verify the necessity of ROS1 or HAT for anthocyanin synthesis. These experiments are technically feasible but have not been systematically conducted, constituting an urgent task for epigenetic regulation research.

Other critical knowledge gaps include: the principal functional PYL receptor in berries at véraison remains unidentified, with functional differentiation and redundancy among the 14 existing PYL members unclear; PP2C substrate specificity and interaction affinities with PYL receptors lack direct determination in grape; the complete SnRK2 substrate spectrum is unknown, and whether it phosphorylates MBW complex members or metabolic enzymes warrants exploration; although the epigenetic basis of environmental memory is supported by Arabidopsis evidence, H3K27me3 dynamics at the VvMYBA locus following high-temperature treatment have not been directly measured; mechanisms establishing and maintaining inter-cultivar epigenetic differences remain unclear—are they determined by genetic programs or shaped by environmental history?

Looking forward, three research directions hold the greatest breakthrough potential. Direction one: epigenetic editing for breeding. If the dual-gating model holds, precision “unlocking” of chromatin at the VvMYBA locus through CRISPR-dCas9 fusion with demethylases or histone acetyltransferases could potentially improve cultivar coloration capacity without altering DNA sequence. This strategy avoids linkage drag inherent to traditional breeding, providing new tools for molecular design breeding. Direction two: decoding and harnessing environmental memory. Understanding how high temperature induces and maintains H3K27me3 repression may reveal new approaches for “training” plants to adapt to climate change. For example, could short-term low-temperature “pre-treatment” before véraison enhance subsequent anthocyanin responses through epigenetic modifications? Such an “epigenetic vaccine” strategy warrants exploration. Direction three: systems biology through multi-omics integration. Combining whole-genome bisulfite sequencing (WGBS), chromatin accessibility profiling (ATAC-seq), histone modification mapping (ChIP-seq), transcriptomics (RNA-seq), and metabolomics (LC-MS) during véraison of a single cultivar or in comparative studies across cultivars would systematically dissect the dynamic coordination of the five-layer network. Machine learning models could integrate multidimensional data to predict cultivar responses and identify regulatory hubs.

This review redefines the role of the epigenetic layer—from passive recorder to active decision-maker, from consequence of transcription to prerequisite for transcription. This paradigm shift applies not only to grape anthocyanins but may represent a universal principle of fruit ripening regulation. As epigenetic regulatory mechanisms are elucidated, we will more profoundly understand how plants integrate genetic programs, developmental signals, and environmental history through chromatin states to achieve precise spatiotemporal-specific gene expression. Regulation of anthocyanin biosynthesis is ultimately a five-layer symphony—each layer is indispensable, with the epigenetic layer serving as conductor, determining the timing, intensity, and persistence of this performance.
